# Supplementary material for: CASC3 promotes transcriptome-wide activation of nonsense-mediated decay by the exon junction complex
Source: Nucleic Acids Res. 2020 Jul 4;48(15):8626–44. doi: 10.1093/nar/gkaa564 (PMC7470949; doi:10.1093/nar/gkaa564)
Supplement: gkaa564_Supplemental_Files [file gkaa564_supplemental_files.zip › Supplementary Figures.pdf]

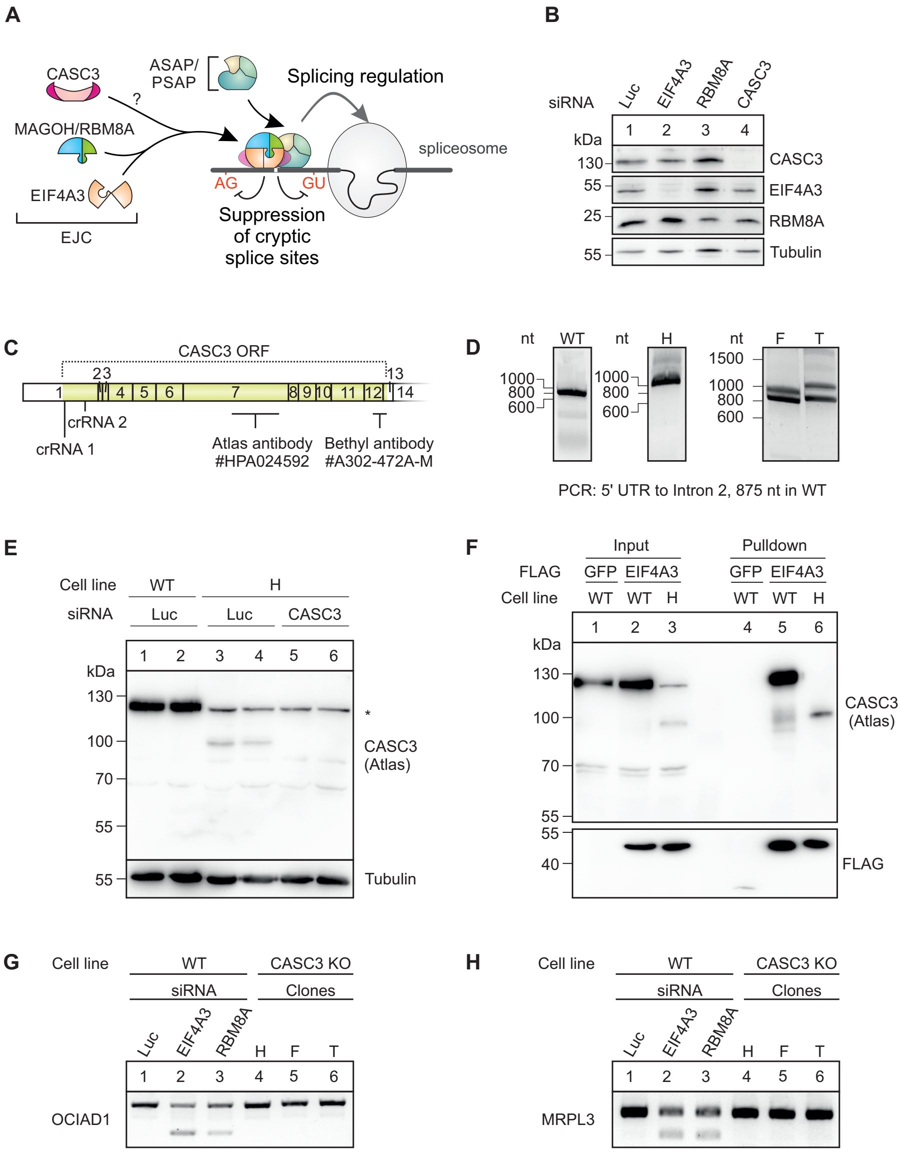

Supplementary Figure 1 - Establishment and validation of CASC3 KO cell lines.

A: Model of splicing regulation by the EJC, modified from Boehm *et al.* (2018).

B: Confirmation of the knockdowns shown in Figure 1A-D by western blotting.

C: Recognition sites of the two antibodies used in this study to detect CASC3. The coding exons 1-13 and a part of the non-coding exon 14 of CASC3 are depicted. The open reading frame (ORF) is marked yellow. The positions of the crRNA sequences to generate the knockout cells are also indicated.

D: Amplification of the genomic CASC3 locus targeted by CRISPR-Cas9 editing in the cell lines H, F and T. The sequences of the insertions were analyzed by cloning and Sanger-sequencing of the respective PCR-fragments and are shown in Figure 1F.

E: Western blot of cell lysates from WT cells and the CASC3 KO cell line H that have been treated with the indicated siRNAs.

F: Co-immunoprecipitation of FLAG-GFP and FLAG-EIF4A3 that were stably expressed in the indicated cell lines. Endogenous CASC3 and the recombinant FLAG proteins are detected by western blotting.

G and H: RT-PCR of transcript isoforms of the genes OCIAD1 (G) and MRPL3 (H) after siRNA-mediated knockdown of the indicated EJC components, Luciferase (Luc) as a negative control and in the CASC3 KO cell lines H, F and T.

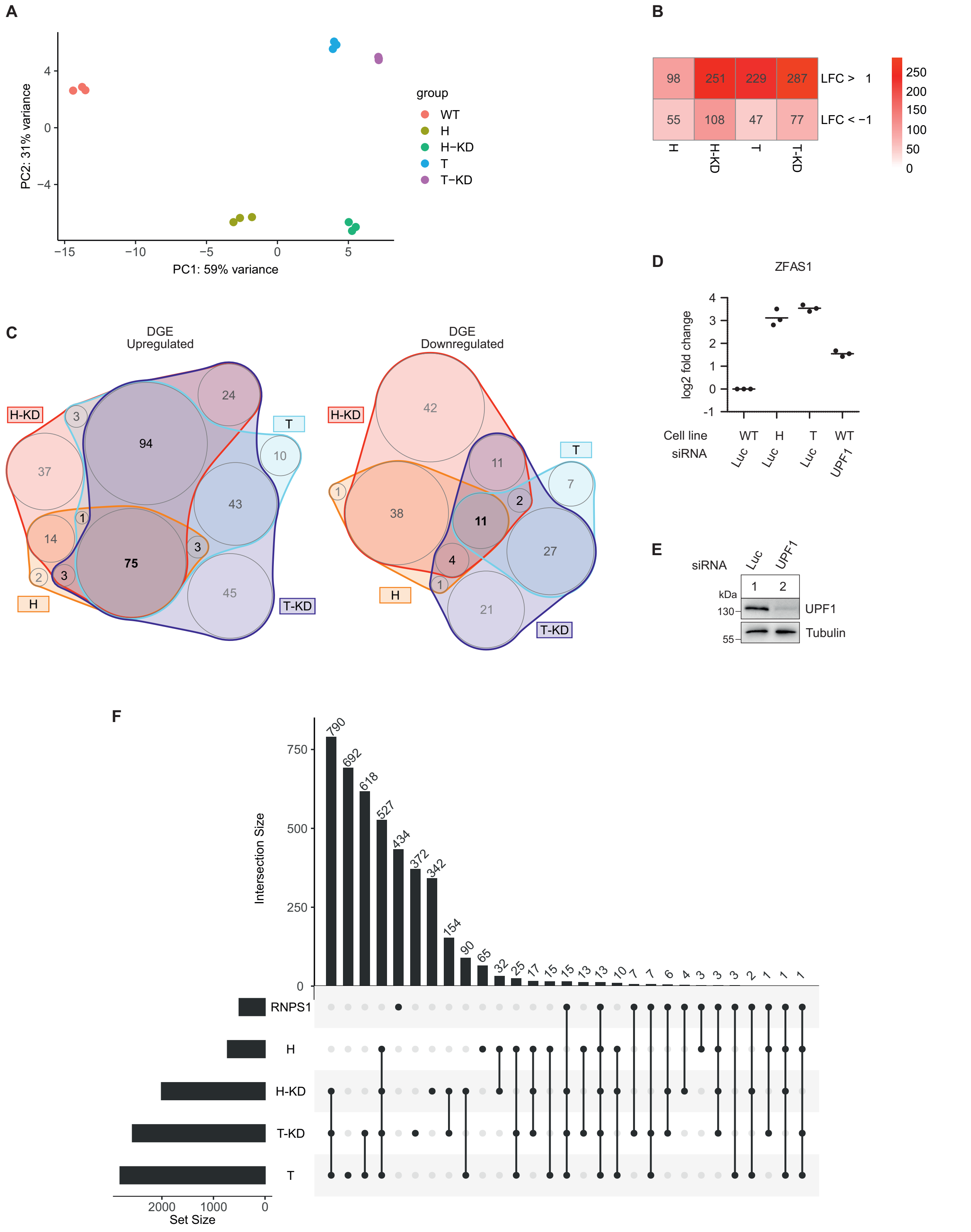

Supplementary Figure 2 - Transcriptome-wide effects of the CASC3 KO.

A: Principal component analysis (PCA) of differential gene expression data.

B: Summary of significantly up- or downregulated genes in the differential gene expression analysis with an absolute log2 fold change (|LFC|) > 1.

C: Overlap of all intersections of differentially expressed genes with an absolute log2 fold change (|LFC|) > 1 in all CASC3 KO conditions shown as nVenn plot.

D: Relative quantification by qPCR of the ZFAS1 transcript in WT cells, CASC3 KO cell lines H and T and WT cells treated with siRNA targeting UPF1. Individual data points and means are plotted (n=3).

E: Confirmation of the UPF1 knockdown by western blotting.

F: Upset plot of intersections of alternatively spliced junctions in the shown conditions.

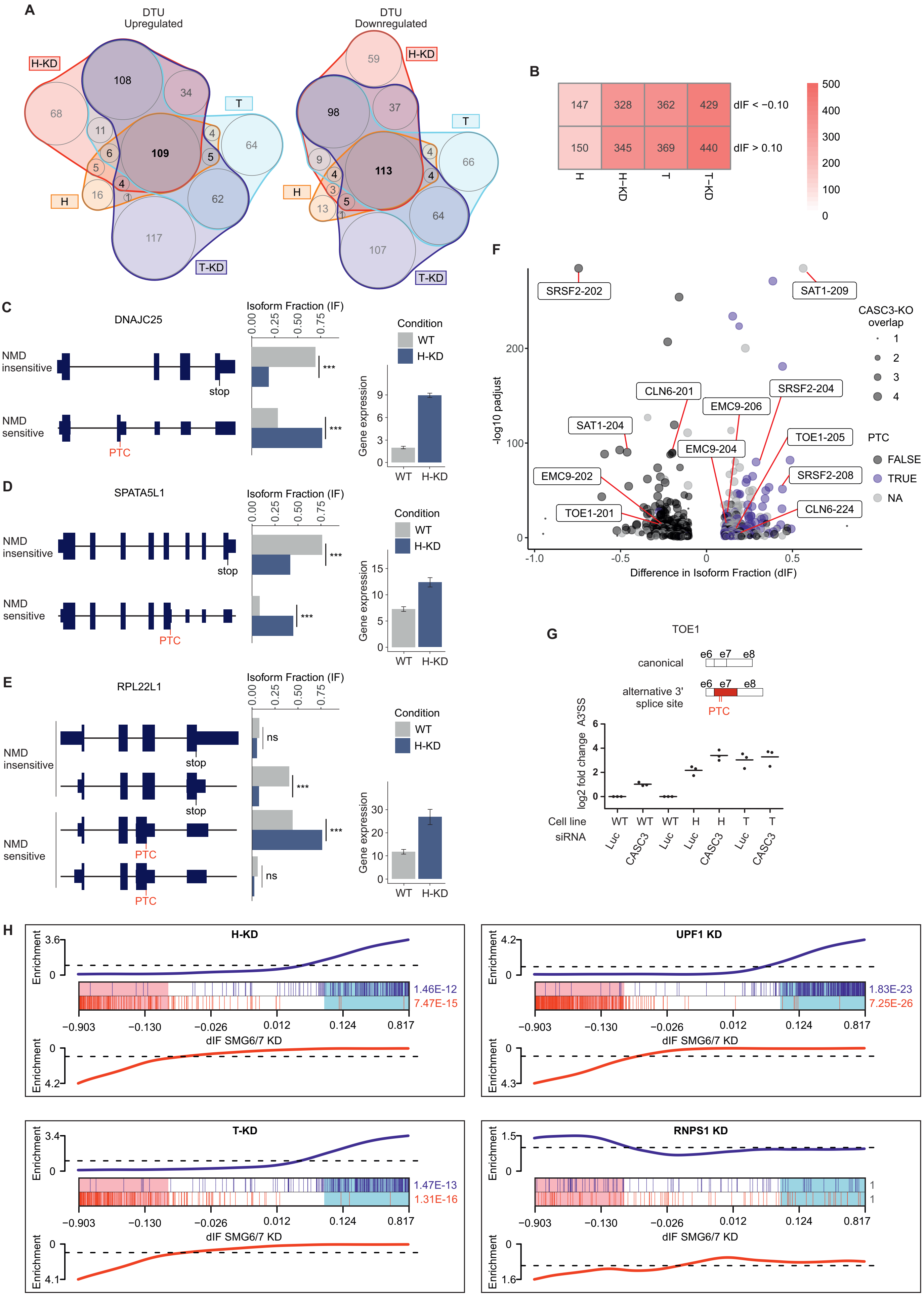

Figure legend on next page

Supplementary Figure 3 - Analysis of differentially expressed transcript isoforms in the CASC3 KO.

A: Overlap of all intersections of transcript isoforms up-or downregulated in IsoformSwitch analysis with an absolute Difference in Isoform Fraction (|dIF|) > 0.1 shown as nVenn plot. DTU: Differential transcript usage.

B: Summary of transcript isoforms up- or downregulated in IsoformSwitch analysis with an absolute Difference in Isoform Fraction (|dIF|) > 0.1.

C-E: Quantification of transcript isoforms from DNAJC25 (**C**), SPATA5L1 (**D)**, RPL22L1 (**E**) by IsoformSwitchAnalyzeR.

F: Volcano plot of IsoformSwitch data for the condition H-KD using overlap from Supplementary Figure S3A as point size definition and PTC status as color definition. Isoforms with no annotated open reading frame are designated as “NA”. Ensembl transcript names are indicated for the genes validated in Figure 3C-E and Supplementary Figure S3G. Difference in Isoform Fraction (dIF) is plotted against –log10 padjust (adjusted p-value).

G: Relative quantification of the schematically depicted transcript isoforms from the gene TOE1 by qPCR in WT cells treated with Luc siRNA as a negative control, WT cells treated with CASC3 siRNAs, CASC3 KO cell line H treated with Luc siRNA, CASC3 KO cell line H treated with CASC3 siRNAs and CASC3 KO cell line T treated with Luc siRNA as well as CASC3 KO cell line T treated with CASC3 siRNAs. Individual data points and means are plotted (n=3).

H: Barcode plots as shown in Figure 3G. The worms represent the relative enrichment of vertical bars in the plot.

A

Significant in WT/CTL, KO/CTL and KO/WT

	WT/CTL	KO/CTL	KO/WT
CWC22	3.92	4.48	0.54
ZC3H11A	3.38	3.00	-0.44
SDE2	3.30	4.02	0.72
CHTOP	3.21	2.63	-0.52
DDX39B	3.18	2.80	-0.42
PPWD1	2.72	3.53	0.69
EIF4G1	1.96	1.21	-0.71
CCDC12	1.78	2.20	0.42
DDX17	1.25	0.95	-0.33

Significant in WT/CTL and KO/WT

CASC3	4.74	0.29	-4.34
UPF3B	3.97	2.45	-1.52

Significant in KO/CTL and KO/WT

USP15	0.10	0.28	0.14
CMBL	0.15	-0.47	-0.62
WARS	0.12	-0.97	-1.08

Significant in WT/CTL and KO/CTL

EIF4A3	6.12	5.50	-0.62
PYCR2	5.00	3.47	-1.57
RBM8A	4.92	4.86	0.02
MAGOHB	4.82	4.82	0.03
ACIN1	4.60	3.78	0.07
PNN	4.49	4.50	-0.01
RNPS1	4.08	3.85	0.13
CRNKL1	4.05	4.57	0.53
THOC5	4.00	3.59	-0.51
ZC3H14	3.93	3.72	-0.16
SAP18	3.92	4.00	0.12
KIF1C	3.91	4.25	0.26
DDX3X	3.88	2.65	-1.25
EIF2A	3.85	2.78	-1.05
CWC27	3.83	4.87	0.45
SRRM2	3.78	3.56	-0.37
CCDC9	3.74	3.58	-0.06
CDC5L	3.68	3.95	0.25
THOC2	3.64	3.09	-0.44
PRPF19	3.55	3.79	0.25
TRA2B	3.53	3.44	-0.12
PLRG1	3.49	3.71	0.28
PRPF4B	3.49	3.85	0.43
PDCD4	3.41	3.07	-0.27
CDC40	3.40	3.87	0.59
PRPF38A	3.34	3.18	-0.16
SNW1	3.33	3.51	0.27
ARGLU1	3.33	3.22	-0.11
SRSF7	3.32	3.54	0.18
SRSF6	3.29	3.26	-0.03
SNRNP40	3.29	3.50	0.23
PAXBP1	3.27	3.32	0.14
SNRNP200	3.27	3.51	0.21
BUD13	3.27	3.49	0.37
PRPF8	3.27	3.51	0.26
DDX23	3.22	3.31	0.11
EFTUD2	3.15	3.34	0.23

Significant in WT/CTL and KO/CTL continued

TFIP11	3.12	3.16	0.10
SART1	3.11	3.21	0.12
XAB2	3.11	3.61	0.54
CFAP20	3.08	2.74	-0.39
PABPN1	3.06	2.75	-0.17
RBM22	3.05	3.42	0.24
MFAP1	3.00	3.08	0.06
CD2BP2	2.99	2.93	0.09
USP39	2.96	3.10	0.09
RBM25	2.96	2.69	-0.13
SRSF1	2.94	2.90	0.01
PRPF3	2.91	3.16	0.14
PRPF6	2.90	3.06	0.23
THOC1	2.90	2.63	-0.29
HIST1H2BN	2.84	2.84	0.35
IK	2.83	2.95	0.09
PRPF40A	2.81	2.59	0.06
HIST2H3PS2	2.77	2.65	-0.25
HIST1H1C	2.74	2.53	-0.33
SMU1	2.73	2.67	0.02
PRPF4	2.73	2.84	0.16
SNRPA1	2.72	3.00	0.24
SF3B2	2.66	2.83	0.13
CTNNBL1	2.65	2.76	0.07
SF3B3	2.52	2.70	0.10
SF3B1	2.51	2.66	0.10
TCERG1	2.51	2.14	-0.39
SF3A1	2.48	2.64	0.14
WBP11	2.46	2.37	-0.15
FNBP4	2.45	2.73	0.31
DHX15	2.38	2.18	-0.14
SNRPB2	2.31	2.49	0.20
SF3A3	2.28	2.46	0.18
RBMX	2.18	1.63	-0.56
SNRPD2	2.15	2.31	0.15
SF3A2	2.08	2.32	0.14
CHERP	1.99	2.03	0.03
SNRPD1	1.90	2.04	0.15
U2SURP	1.90	1.72	-0.13
BCLAF1	1.90	2.19	0.25
LUC7L3	1.86	1.94	0.26
HNRNPM	1.79	1.59	-0.17
XRCC6	1.76	1.26	-0.33
XRCC5	1.74	1.36	-0.31
THRAP3	1.60	2.25	0.65
DHX38	1.59	1.74	0.11
SRPK1	1.58	1.36	-0.18
PRKDC	1.56	1.55	0.07
SRRT	1.56	1.41	-0.22
SARNP	1.28	1.09	-0.34
EIF3G	1.20	0.79	-0.51
SAFB	1.18	0.89	-0.32
TAF15	1.01	0.91	-0.09
DDX5	0.96	0.89	-0.03
SNRPD3	0.70	0.98	0.26
USP9X	0.53	0.72	0.01

Significant in WT/CTL only

PYCR1	4.45	2.29	-1.83
PPIG	3.51	3.38	0.21
HIST1H4A	2.73	2.69	-0.06
RPL8	2.41	1.86	-0.66
DHX8	2.28	2.57	0.60
CDK11B	2.24	2.82	0.57
RPL7A	2.20	1.40	-0.76
RPL13	2.11	1.48	-0.77
RPL6	1.99	1.18	-0.91
RPL7	1.87	0.82	-0.71
PARP1	1.86	2.03	0.08
EIF3A	1.82	0.88	-0.96
EIF3C	1.81	0.93	-0.98
RPS26	1.77	0.55	-1.23
RBM17	1.77	1.43	-0.40
CSNK2A1	1.63	1.08	-0.40
RPL34	1.56	0.83	-0.66
RPS8	1.54	0.82	-0.61
RPS9	1.52	0.96	-0.63
RPS14	1.51	0.85	-0.74
RPS4X	1.46	0.77	-0.61
RPS7	1.45	0.78	-0.57
RPS3A	1.43	0.76	-0.68
EIF3I	1.39	0.67	-0.87
RPS20	1.29	0.70	-0.55
RPS25	1.29	0.69	-0.63
RPS3	1.28	0.66	-0.66
RPS16	1.28	0.65	-0.66
RPSA	1.26	0.62	-0.68
U2AF2	1.26	1.39	-0.04
RPS18	1.24	0.53	-0.75
GNB2L1	1.20	0.60	-0.65
LUC7L2	1.19	1.00	-0.10
SNRNP70	1.01	0.66	-0.36
KHDRBS1	0.89	0.25	-0.68
IRS4	0.75	-0.44	-1.19
UBB	0.62	0.25	-0.44
FUS	0.44	0.74	0.30
NONO	0.44	-0.14	-0.59
SFPQ	0.37	-0.07	-0.47
NSRP1	0.36	0.74	0.43
GEMIN5	0.21	-0.07	-0.27
HNRNPA1	0.14	-0.05	-0.22
ANKFY1	-0.32	0.21	0.49

Significant in KO/CTL only

AQR	4.85	4.10	0.55
SON	3.14	3.18	0.02
RSRC1	3.45	3.04	-0.12
SFSWAP	3.20	2.87	-0.04
RBM39	2.11	2.25	-0.02
FAT3	1.69	1.78	0.00
SNRPN	0.45	0.92	0.35
HSPA8	0.44	0.34	-0.07
HSPH1	0.34	0.33	-0.14
HSPA1B	0.69	0.31	-0.38
SMC1A	0.28	0.17	-0.12
HDAC6	0.69	-0.56	-0.72

Figure legend on next page

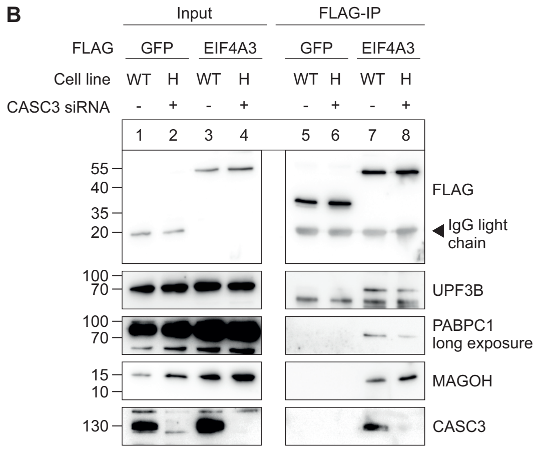

Supplementary Figure 4 - Protein interactions of EJC core and peripheral factors in CASC3 KO cells.

A: All targets are listed that are shown in the volcano plots in Figure 4. The log2 fold changes of the WT/CTL (first column), KO/CTL (second column) and KO/WT (third column) comparisons are shown. The color of points corresponds to the volcano plots in Figure 4. Full results are shown in Supplementary Table S2.

B: Co-immunoprecipitation of lysates obtained from the indicated cell lines stably expressing either FLAG-emGFP or FLAG-EIF4A3. Before harvesting the cells were treated with 50 µg/ml of cycloheximide to inhibit translation. No RNase was used during lysis to maintain RNA-protein interactions.

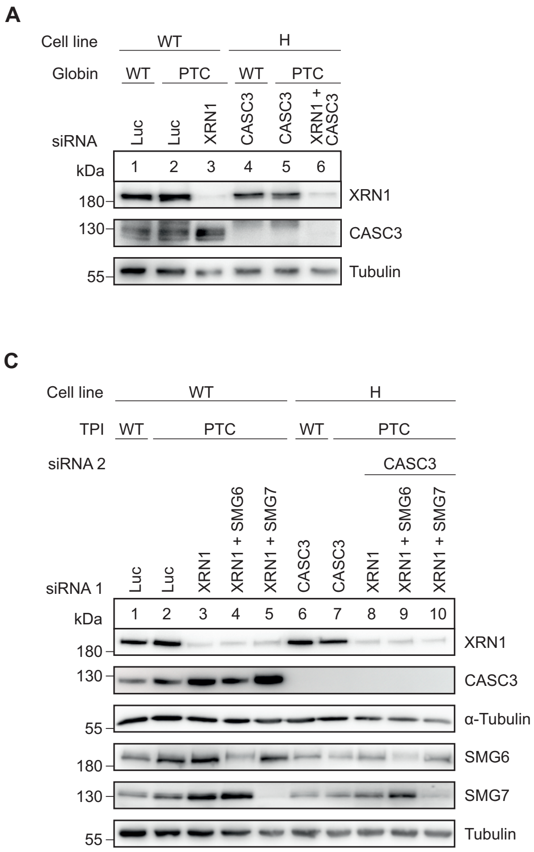

Supplementary Figure 5 - Confirmation of knockdowns performed for NMD reporter assays.

A: Western blot of samples shown in Figure 5B.

B: Western blot of samples shown in Figure 5D.

C: Western blot of samples shown in Figure 5F.

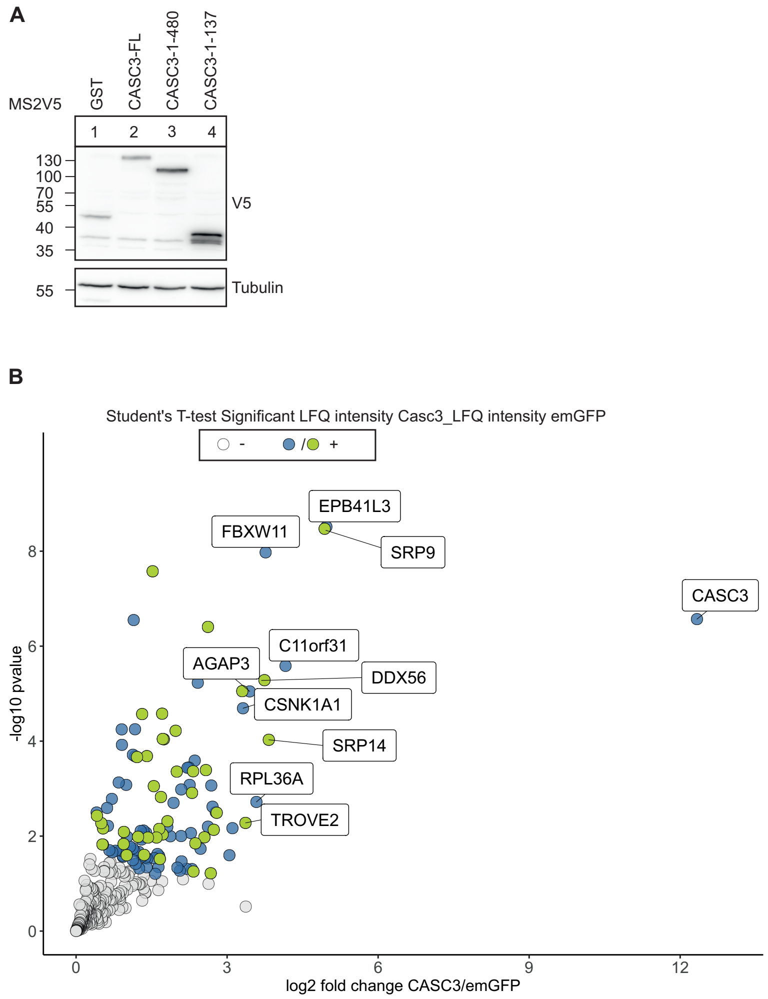

Supplementary Figure 6 - Interactome of the CASC3 N-terminus.

A: Western blot of samples shown in Figure 6B.

B: Volcano plot of mass spectrometry-based analysis of the interaction partners of the CASC3 N-terminus (first 137 amino acids) in WT cells. Only positively enriched factors are plotted. The colored points indicate targets that are significant in the respective comparisons after one-sample t-testing. The top 10 significant hits are labeled. Significant hits that are annotated as „RNA binding“ (GO:0003723) in the Gene Ontology domain „molecular function“ are shown in green.
